# Association Between Psychological Distress and Coping Styles in Family Caregivers of People with Intellectual Disability or Chronic Mental Disorder in Mongolia

**DOI:** 10.3390/nursrep14040257

**Published:** 2024-11-15

**Authors:** Delgermaa Sendmaa, Namuun Ganbaatar, Orgilmaa Regzedmaa, Erdenetuul Nuden, Enkhtuul Chuluun, Sundui-Yanjmaa Luvsangenden, Gankhuyag Gochoosuren, Dolgorjav Myagmarjav, Oyungoo Badamdorj, Khishigsuren Zuunnast, Myagmartseren Dashtseren, Naranbaatar Nyam, Fiona Nolan

**Affiliations:** 1 Department of Adult Health Nursing, School of Nursing, Mongolian National University of Medical Sciences, Ulaanbaatar 14210, Mongolia; delgermaa.s@mnums.edu.mn (D.S.); orgilmaa@mnums.edu.mn (O.R.); enkhtuul.ch@mnums.edu.mn (E.C.); sundui-yanjmaa@mnums.edu.mn (S.-Y.L.); 2 Department of Physical and Occupational Therapy, School of Nursing, Mongolian National University of Medical Sciences, Ulaanbaatar 14210, Mongolia; namuun@mnums.edu.mn; 3 Mongolian National Centre of Mental Health, Ulaanbaatar 13381, Mongolia; erdenetuul@ncmh.gov.mn; 4 Department of Fundamentals Nursing, School of Nursing, Mongolian National University of Medical Sciences, Ulaanbaatar 14210, Mongolia; gankhuyag@mnums.edu.mn (G.G.); dolgorjav@mnums.edu.mn (D.M.); naranbaatar.n@mnums.edu.mn (N.N.); 5 School of Nursing, Mongolian National University of Medical Sciences, Ulaanbaatar 14210, Mongolia; 6 Department of Mental Health, School of Medicine, Mongolian National University of Medical Sciences, Ulaanbaatar 14210, Mongolia; khishigsuren@mnums.edu.mn; 7 Department of Family Medicine, School of Medicine, Mongolian National University of Medical Sciences, Ulaanbaatar 14210, Mongolia; myagmartseren@mnums.edu.mn; 8 North London NHS Foundation Trust, St. Pancras Hospital, 4, St. Pancras Way, London NW1 0PE, UK; fiona.nolan3@nhs.net

**Keywords:** caregiver, intellectual disability, severe mental illness, coping, psychological distress

## Abstract

Around the world, family caregivers are an important source of support for people with intellectual disability (ID) and for those with severe mental disorder (SMD), although the level of support can be influenced by the culture and government healthcare systems in each country. However, there is little evidence about the mental health and coping mechanisms of these caregivers in low-income countries. To address this need, we aimed to elicit whether there are potential links between coping style, mental health, and perceived burden experienced by this group, using a sample from a central Asian upper middle-income country. Methods: We recruited 120 participants, of which 60 were caregivers of people with ID and 60 of people with SMD. All participants were recruited from Ulaanbaatar, the capital city of Mongolia, Central Asia, and were asked to complete of the Depression, Anxiety and Stress Scale (DASS) and the Coping Orientation to Problems Experienced inventory scale (COPE). Multiple regression analyses were used to investigate associations between these measures. Results: We found that DASS scores were significantly higher among the sample of caregivers of individuals with ID than in those of SMD. Mental and behavioral disorders were associated with higher DASS scores in the sample of caregivers of those with SMD. Good coping styles, indicated by higher scores in the COPE, were associated with increased age in caregivers of individuals with ID. Conclusion: Although overall the carers of people with SMD appeared to have better active coping skills and better acceptance of the caring role, they demonstrated comparatively high levels of stress. This study was not registered.

## 1. Introduction

Chronic psychiatric disorders affect many people worldwide, leading to numerous negative social and psychological impacts [1]. The Global Burden of Diseases, Injuries, and Risk Factors Study (GBD) 2019 reported that mental disorders are leading causes of burden worldwide, with prevalence estimates [2]. In addition, the global number of disability-adjusted life years (DALYs) due to mental disorders increased from 80.8 million to 125.3 million between 1990 and 2019 [2]. Moreover, it is estimated that 1 billion people have a mental health disorder worldwide [3], and that 107.6 million have intellectual disability (ID), which is a prevalence of 4.39% for MH disorder and 1.74% for ID [2,4].

People with intellectual disabilities (ID) have a higher risk of mental health conditions than people without ID. A meta-analysis of articles published from 1985 to 2018 found that 33.6% of people with ID had a mental health condition [5].

Family members are important caregivers for people with ID and psychiatric illnesses [6]. Four studies conducted between 2014 and 2016 in Singapore Jordan, Greece, and India, with a combined participant sample of 1100, found that these groups experienced a greater prevalence of depression and anxiety and higher levels of perceived stress than the general populations in the respective countries. They also reported a comparatively poorer quality of life and more frequent attendance of mental health outpatient clinics [7,8,9,10]. An earlier study conducted in the USA found that mortality risks amongst caregivers were 63% (relative risk [RR], 1.63; 95% confidence interval [CI], 1.00–2.65) higher than non-caregivers [11]. Chang et al., (Singapore, 2016) recommended that caregivers need help to cope with physical, mental, and emotional distress [7].

Further studies conducted in the USA (Phelan, Bromet, and Link, 1998) and Ethiopia, (Shibre et al., 2001), with sample sizes of 195 and 178, respectively, found that 10% of carers of people with mental illness reported being avoided by members of the public and 20% to 30% reported low self-esteem linked to having a relative with mental illness only rather than ID and severe mental disorder (SMD) [12,13].

The theoretical framework for the current study was based on a transactional model of stress and coping, which was developed by Lazarus and Folkman in the USA, 1984. This model incorporates principles based on two states: the first of which entails cognitive response to stressful situations and the second the response to these situations [14]. Accordingly, when parents of children with ID are not equipped with skills to cope effectively with their caring burden, one study has suggested that they seem to be strongly affected by emotional distress, which the authors proposed may be due to lack of engagement in activities which promote better mental health [15].

Developmental disability (DD) is a neurological abnormality with onset in childhood that is associated with long-term developmental and neurological and deficits, including intellectual disabilities, that affect physicality, learning, language, and behavior [16]. A study conducted by Marquis et al. (Canada, 2020) found that caregivers of children with DD demonstrated mild to moderate symptoms of depression and anxiety, and increased risk of the mental health [17,18]. This study (in addition to a previous paper by the same authors in 2019) suggested that problem-oriented and active emotional (positive) coping strategies which are used by carers of people with SMD might be beneficial to parents of children with ID and DD [18]. A further USA study with a sample of 20,013 caregivers of children with DD found that having a realistic expectations of the child’s disability and acceptance of the situation helped them cope [19].

Murphy et al. (2007) found that religious beliefs could potentially give a feeling of hope, harmony, and strength to caregivers for children with disabilities, assisting in acceptance and adaptation. The authors defined ‘religious coping’ as ‘strategies that include appraising a secure relationship with a benevolent higher power, believing that there is meaning in life, and seeking support from temple/church members’ [20].

The life expectancy of people with ID has increased in developed countries due to better management and medical advances [21]. However, the lack of adequate funding to support public healthcare systems in developing countries has been demonstrated to negatively impact on quality of life, thus increasing the prevalence of mental disorders and caregiver burden and decreasing access to treatment [6].

The Convention on the Rights of Persons with Disabilities (CRPD) emphasizes that member states must improve their understanding of legal capacity while respecting the rights and preferences of individuals with mental and intellectual disabilities. Achieving this goal necessitates systemic changes within professional cultures and sufficient funding for mental health national and local services [22]. However, the understanding of a social model including social equality for intellectual and developmental disabilities remains unclear [23].

Therefore, formal support for people with ID in developing countries is severely lacking. These support avenues include but are not limited to psychosocial rehabilitation, community-based services, and support for families. Evidence around socioeconomic issues, clinical care needs, and caregiver burden related to people with ID and SMD in developing countries, including Mongolia, is very limited. The impact of this role on the caregiver’s mental health and potential relationship between their coping mechanisms and any psychological distress they may experience remains unclear. This lack of evidence has been highlighted by the previously referenced study by Marquis. et al. (Canada, 2019) [18,24].

We aim to explore and compare associations between coping styles, perceived burden, and the mental health of caregivers in two groups comprising those caring for people with ID and those caring for people with SMD.

## 2. Materials and Methods

This is an observational, cross-sectional study. We used the Strobe checklist (Strengthening the Reporting of Observational studies in Epidemiology) to assess the quality of our study. This is found in Appendix A.

### 2.1. Setting

Participants for the study were recruited from caseloads of primary healthcare centers in all 8 districts in the capital city Ulaanbaatar, Mongolia. The overall population of the country is 3,457,548, of which about 2 million live in Ulaanbaatar [25]. A survey by the United Nations Economic and Social Commission for Asia and the Pacific indicated an overall prevalence of disability in Mongolia of 3.9% (108,071 of the total population of 3,057,778 in 2015) [26]. Mental and intellectual disabilities (including people with both ID and SMD) comprised 19.3% (20,857) of those 108,071 individuals with disability or 0.1% of the total population [26].

### 2.2. Participants

A total of 3268 people with SMD (n = 2625) and ID (n = 643) met the study inclusion criteria from the 8 primary care centers across the 8 districts, and outpatients of the National Center of Mental Health of Ulaanbaatar. Of these, 653 were identified as having caregivers and from those, the researchers contacted a sample of 160, selected according to the methods outlined above, of which 80 were in the ID group and 80 in the SMD group.

**Figure 1:**
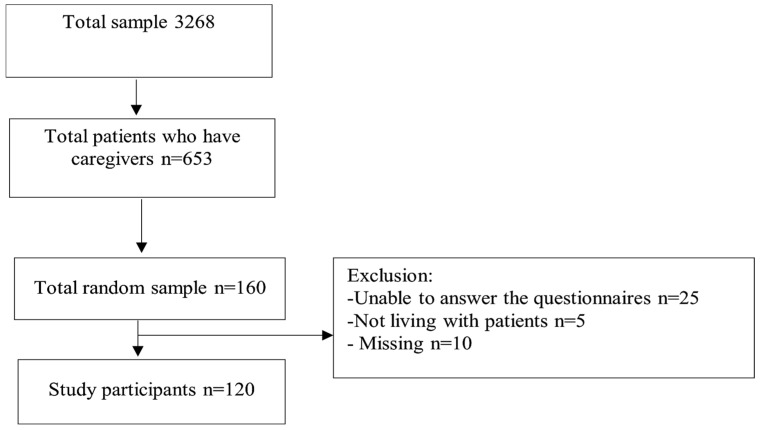
Study flow diagram.

From the total 160 potential participants who were contacted, 5 were not eligible from the SMD group, as we found they were not living with the individual, and 25 were unable to complete the questionnaires. Ten were excluded due to missing data. The study flow diagram in Figure 1 represents our 75% response rate, representing 18.7% of the total 653 originally identified.

### 2.3. Ethical Approval

The Institutional Ethics Committee of the Mongolian National University of Medical Sciences (Ulaanbaatar, Mongolia) approved this study (8 March 2019).

Identifying participants and inclusion criteria.

Recruitment of participants.

A list of all individuals with diagnoses of SMD and ID was obtained by the study researchers from each primary healthcare center’s managers and psychiatrists.

For the purposes of this study, participants in the SMD group comprised only those with diagnoses of mood disorders, schizophrenia, and organic personality disorders who had been receiving treatment for mental disorders for one year or longer. Participants in the ID group comprised only those with moderate or severe ID. Those with mild forms of ID were excluded. Our study included those who met the inclusion criteria and identified people with carers through case note analysis. We defined caregivers as anyone who undertook a caregiving role and was living with the identified person with ID or SMD.

We selected a sample from the total identified number of carers using a method of random sampling.

### 2.4. Method of Approach and Consent

We did not obtain consent from people with ID or SMD who were identified as receiving care from primary healthcare services to approach their caregivers. We instead contacted caregivers directly to ask for their consent to be included in the study. This method was approved by the local research ethics committee.

Caregivers who agreed to participate in the study were asked by the researchers to provide written consent. This was stored separately to the results from the completed questionnaires to preserve the anonymity of the responses.

### 2.5. Measurements

We used a quantitative survey method for data collection, which was completed in face-to-face interviews by participants with assistance from the study researchers.

The questionnaires included three specific sections outlined below and took on average 30 min to complete.

1. This section comprised socio-demographic questions including age, gender, education, family history of the care giver, caregiving period, amount of government financial support through disability allowance, and welfare assistance.
2. Depression, anxiety, and stress scale (DASS) [27]. This is a 42-item questionnaire to assess anxiety, depression, and stress. A higher score indicates higher levels of depression, anxiety, and stress.
3. The Coping Orientation to Problems Experienced (COPE) Inventory scale [28], which is a multidimensional coping inventory to evaluate how people react to stress. The scale contains 60 items, with 13 subscales (four items on each scale, rated on a 4-point Likert scale) which assess conceptually distinct aspects of coping. These 13 subscales are (1) active coping, (2) planning, (3) suppression of competing activities, (4) restraint coping, (5) seeking of instrumental social support, (6) seeking of emotional social support, (7) positive reinterpretation, (8) acceptance, (9) denial of competing activities, (10) religious coping (11) focus on and venting of emotions, (12) behavioral disengagement, (13) mental disengagement [29].

A higher score indicates more effective use of each identified coping skill.

Both the DASS and COPE scales were translated into Mongolian language using a modified version of the Brislin method (1970), which comprises forward and back translation independently by two translators, and then review of the translation by a team of experts in the field. We analyzed Cronbach’s Alpha coefficient of internal reliability for pre- and post-assessment, which were 0.97 and 0.93, respectively, for DASS. Furthermore, for the COPE inventory scale, the Cronbach’s Alpha coefficients of internal consistency were 0.95 and 0.98, respectively. However, we agreed to note any issues with the questionnaires that arose during the study. This was the first time either of the measures had been used in a Mongolian population.

Study sample: The study was conducted between December 2019 to June 2020.

### 2.6. Statistical Analysis

The Statistical Package for R studio (R 4.2.2 version) was used for analyses [30]. Quantitative variables were described as mean and standard deviation, and categorical variables were described as number and percentages. We used Spearman’s correlation test to determine any associations between depression, stress, anxiety, coping styles, and age.

We also used independent sample t-tests and chi-square tests to compare the socio-demographic data between the ID and SMD groups, and multivariate analyses of variance (MANOVA) to examine the caregiver’s differences in depression, anxiety, and stress symptoms.

Chi-square tests were also used to compare the differences in the use of coping styles by the caregivers, and multiple regression analysis was used to investigate associations between the four coping styles and depression and stress symptoms, with the significance level set at 0.05.

## 3. Results

Socio-demographic characteristics of age, gender, and educational achievement between both groups are shown in Table 1. There were statistically significant differences in the age and education levels, with the average age of the caregivers in the ID group being of 37.66 years (SD = 13.62), compared to 45.23 years (SD = 12.72) in the SMI group. The numbers of men and women were equivocal in each group and between the groups (Table 1).

A comparison of the levels of depression, anxiety, and stress symptoms of the ID and SMD groups as measured by the DASS are reported in Table 2.

The depression scores of the SMD group ranged from 0 to 42 (mean = 13.7; SD = 5.85) and the stress scores ranged from 0 to 42 (mean = 12.06; SD = 5.37).

These were lower than those in the ID group, where depression scores ranged from 9 to 42 (mean = 41.73; SD = 10.81), in which 6.66% (n = 4) had severe symptoms and 93.3% (n = 56) had extremely severe symptoms. Stress scores ranged from 0 to 42 (mean = 40.66, SD = 10.94). Further, 23.4% of the ID group (n = 14) had severe symptoms and 70% (n = 42) had extremely severe symptoms (Table 2).

Table 3 shows the results of a comparison between participants’ coping styles.

There was no significant difference between the types of coping styles engaged with: positive reinterpretation and growth, religious coping, and behavioral disorder.

However, a significant difference was shown in the mental disorder of caregivers with ID and caregivers with SMD (F = 15.15, *p* = 0.001) (Table 3).

Table 4 shows the results of the correlation of coping style, psychological status levels, and age of caregivers of individuals with intellectual disabilities. Positive significant correlations were observed between depression (r = 0.835, *p* < 0.001), anxiety (r = 0.826, *p* < 0.001), and stress results (r = 0.953, *p* < 0.001).

The results of positive reinterpretation and growth were significantly positively correlated with mental disengagement (r = 0.636, *p* < 0.001), religious coping (r = 0.545, *p* < 0.001), behavioral disengagement (r = 0.514, *p* < 0.001), and age (r = 0.242, *p* < 0.05). Religious coping was positively correlated with behavioral disengagement (r = 0.587, *p* < 0.001) and age (r = 0.237, *p* < 0.05) (Table 4).

The results of the association between coping styles and psychological status with depression and anxiety are shown in Table 5. The positive reinterpretation and growth coping style was inversely associated with depression and stress in both groups. Further, religious coping and behavioral coping were positively associated with depression and stress (Table 5).

## 4. Discussion

We found that caregivers of people with ID reported significantly higher levels of depression, anxiety, and stress than those of people with SMD in our samples. The ID carers may therefore be at risk of experiencing psychological distress. Our findings replicate those from two studies conducted in India and Pakistan, which reported that caregivers of individuals with ID faced significantly higher levels of psychological distress than caregivers of people with other mental disorders [31,32]. The impact on the family carer’s psychological status may relate to the severity of the intellectual disability and dependency. Therefore, constant assessment of the stress, depression, and burden of caregivers may help to adapt and overcome the burden.

Potential contributory factors to the higher scores in the ID group on the DASS may include concerns regarding their relative’s long-term welfare [33]. Parental stress negatively affects not only caregiving, but also parental mental and physical health [34,35].

A study by Miodrag N. et al. [34] found that mothers with children with developmental disabilities are prone to high stress, negatively impacting their well-being. Therefore, unresolved stress affects their ability, decreasing the quality of parent–child relationships, causing poor psychological health, and increasing anxiety and depression [36]. Moreover, the transactional effect on the relationship between parenting stress and child caregiving burden remains unclear. These effects are essential to coping with stress and family adaptation [37]. Positive-oriented coping skills can be adapted through diverse support from the community, including positive family relationships and reducing psychological stress [38].

The stress process models [39] expose that coping with a particular type of stress helps to overcome psychological distress and becomes relevant [40].

Prior research has reported that a positive illusion can influence mental health through cognitive adaptation [41]. However, the mechanism of these psychological circumstances is less known. Cognitive adaptation has a beneficial role on mental health, which is affected by motivational processes [42]. However, we did not find any significance between psychological status and positive coping. In contrast, we found that use of the coping style of positive reinterpretation and growth inversely correlated with stress and anxiety, and that it helps reduce the ongoing stress’s impact, resulting in lower symptoms of depression among caregivers [32].

Moreover, our findings indicate that mental and behavioral disorders were positively associated with depression, anxiety, and stress levels in the SMD group. This may be attributed to the greater adoption of coping behavioral disengagement in the SMD group.

We also found that most respondents had religious beliefs, and that these were related to positive reinterpretation and growth coping. Religious feelings may therefore perform a significant role in helping family members cope with the stress of caring for a relative with an ID [32]. Our findings echo whose found in two other studies of caregivers of patients with mental illness, which indicated that stronger religious beliefs correlated with less depression and better self-esteem and self-care [43,44].

Furthermore, with increase in age, the positive coping styles tended to be higher in our sample of caregivers of individuals with ID. This may relate to the adaptation and positive hope of caregivers in the improvement of their child. Therefore, after accepting that a child has an ID, the initial focus of a caregiver’s physical and emotional energy may center around training, skills development, and a positive hope for improvement, echoed by the findings of Majumdar M et al. [45].

A patient with mental illness is hospitalized for longer at certain times in their life compared to other patients, and in some cases, complications may occur [46]. Nursing staff spend more time with patients and families due to their job role [47]. They may face more mental distress related to the environment and workloads [48]. Therefore, a highly trained nurse working with mental health disorder patients would feel more confident and less distressed.

Moreover, mental health nurses may use the above scales to assess and recognize the early stages of a family members’ psychological distress that need to be improved, and they can develop individual interventions through coping and adaptation to the situations. To optimize the management of ID and SMD patients, a multidisciplinary, team-based, and family-centered approach is needed. Therefore, improving the quality of specialists requires collaboration between care professionals, policymakers, and organizational managers [49].

Furthermore, in Mongolia, addressing issues related to public perception, social environment, government support, and systematic family services for caregivers of people with intellectual disabilities and severe mental disorders is required. The government and non-governmental organizations must provide special professional assistance to create community-based rehabilitation day care and inpatient care for people with intellectual disabilities.

In addition, developing and implementing various service programs aimed at empowering caregivers with care and nursing, providing support and counseling information, and locally establishing a specialized center for the psychological counseling of caregivers will reduce the burden of caregivers, provide psychological support, and overcome difficulties.

### Limitations and Recommendations

Use of the DASS and COPE measures in the Mongolian language was innovative and may have produced findings that had previously not been explored. However, the validity of the translated forms of the measures has not yet been confirmed. We recommend that this is the next stage in the future use of these measures in Mongolian populations.

The sample was recruited from all areas in the capital city of Mongolia, using existing relationships and cooperation within healthcare systems, enabling this complex study to be completed without any funding. However, we recognize that the sample size may not have been representative of the overall population of caregivers of people with these diagnoses in Mongolia.

Secondly, there was a significant difference in ages between the groups. Age may influence a caregiver’s experience, coping skills, and perceived burden. So, a broader study with increased numbers across all age groups would be useful. The study was designed, implemented, analyzed, and reported by professionals only, without input at any stage from people with SMD or ID, or from their caregivers. Co-production at all stages would benefit future studies, and this study can be seen as the first step towards creating a body of evidence that is relevant to these groups.

The process of obtaining consent from participants, whilst implemented according to the requirements of the local research ethics committee, did not require consent for the researchers to contact the caregivers of individuals receiving treatment for diagnoses of SMD and ID. This ethical consideration should be included in future studies, and more emphasis should be placed on the provision of information about prospective research to people with these diagnoses, and their caregivers, prior to agreement to participate.

Finally, comprehensive demographic and clinical data (including age of patient, duration of illness, educational level of caregivers) were not gathered on the people with ID or SMD, nor were data on total hours spent caring per week on average, or hours of assistance received from other family members, friends, or professionals. This information may have an impact on the outcomes measured in our study and should be included in future research.

## 5. Conclusions

Although overall the carers of people with SMD appeared to have better active coping skills and better acceptance of the caring role, they demonstrated comparatively high levels of stress. Moreover, caregiver-focused psychotherapy can help improve future psychological well-being, reduce burden, and overcome stress. Therefore, further clinical research using a multidisciplinary approach in similar low-income countries is needed to explore this important issue.

## Figures and Tables

**Table 1:** Characteristics of caregivers (n = 120).

Variables	Intellectual Disability (n = 60)	SMD (n = 60)	*p*
Age, years	37.66 (13.62)	45.23 (12.72)	<0.001
Gender			1.00
Men	29 (48.4%)	28 (46.7%)	
Women	31 (51.6%)	32 (53.3%)	
Educational level			<0.001

Note: SMD, severe mental disorders. Continuous variables described as mean (SD), categorical variables. Described as number (percentage).

**Table 2:** Comparison of caregiver’s psychological status levels (n = 120).

Variable	Group	Normal	Mild	Moderate	Severe	Extremely Severe	Total Score	*p*
Depression	ID	0	0	0	4 (6.66)	56 (93.3)	41.73 (10.81)	0.049
	SMD	17 (28.3)	12 (20)	23 (38.3)	7 (11.8)	1 (1.6)	13.7 (5.85)	
Anxiety	ID	0	0	1 (1.6)	11 (18.4)	48 (80)	23.75 (5.66)	0.131
	SMD	18 (30)	12 (20)	21 (32)	8 (13.4)	1 (1.6)	9.5 (4.08)	
Stress	ID	0 (0)	1 (1.6)	3 (5)	14 (23.4)	42 (70)	40.66 (10.94)	0.016
	SMD	38 (63.3)	14 (23.3)	8 (13.4)	0	0	12.06 (5.37)	

Note: SMD, severe mental disorders; ID, intellectual disability. Group values described as number (percentage); total score described as mean (SD).

**Table 3:** Comparison of coping styles between caregivers of individuals with intellectual. Disability (n = 60) and severe mental disorders (n = 60).

Coping Styles	ID (n = 60)	SMD (n = 60)	F	*p*
Positive reinterpretation and growth	9.28 (2.73)	9.08 (2.08)	0.203	0.653
Mental disengagement	7.38 (2.32)	8.9 (1.91)	15.15	0.001
Religious coping	8.05 (2.88)	8.31 (2.01)	0.345	0.558
Behavioral disengagement	7.55 (2.29)	8.15 (1.58)	2.779	0.098

Data shown as mean (SD); ID, intellectual disability; SMD, severe mental disorders.

**Table 4:** Correlation of psychological status, coping styles, and age of caregivers of individuals with intellectual disability (n = 60) and SMD (n = 60).

	Variables		1	2	3	4	5	6	7	8
1	Depression	ID		0.835 ***	0.953 ***	0.045	−0.001	−0.171	0.084	−0.11
		SMD		0.850 ***	0.881 ***	0.111	0.345 **	0.025	0.314 **	0.029
2	Anxiety	ID	0.835 ***		0.826 ***	0.016	0.029	−0.128	0.025	−0.08
		SMD	0.850 ***		0.778 ***	0.072	0.383 **	−0.022	0.242 *	−0.016
3	Stress	ID	0.953 ***	0.826 ***		0.016	0.025	−0.155	0.099	−0.139
		SMD	0.881 ***	0.778 ***		0.011	0.289 *	0.059	0.273 *	0.078
4	Positive reinterpretation and growth	ID	0.045	0.016	0.016		0.636 ***	0.545 ***	0.514 ***	0.242 *
		SMD	0.111	0.072	0.011		0.298 *	0.114	0.288	−0.206
5	Mental disengagement	ID	−0.001	0.029	0.025	0.636 ***		0.592 ***	0.809 ***	0.175
		SMD	0.345 **	0.383 **	0.289 *	0.298 *		0.556 ***	0.602 ***	0.182
6	Religious coping	ID	−0.171	−0.128	−0.155	0.545 ***	0.592 ***		0.587 ***	0.237 *
		SMD	0.025	−0.022	0.059	0.114	0.556 ***		0.437 ***	0.018
7	Behavioral disengagement	ID	0.084	0.025	0.099	0.514 ***	0.809 ***	0.587 ***		0.022
		SMD	0.314 **	0.242 *	0.273 *	0.288 *	0.602 ***	0.437 ***		0.01
8	Age	ID	−0.11	−0.087	−0.139	0.242 *	0.175	0.237 *	0.022	
		SMD	0.029	−0.016	0.078	−0.206	0.182	0.018	0.01	

Note: BD, behavioral disengagement; MD, mental disengagement; SMD, severe mental disorders; PR and G: positive reinterpretation and growth; RC: religious coping. * *p* < 0.05; ** *p* < 0.01; *** *p* < 0.001.

**Table 5:** Multiple regression analyses for variables predicting depression and stress symptoms in overall. Caregivers (n = 120).

Variable	Depression		Stress	
	B	SE	B	SE
Positive reinterpretation and growth	−2.56	9.26	−0.39	9.46
Mental disengagement	0.46	0.69	−0.31	0.71
Religious coping	1.10	0.82	1.25	0.84
Behavioral disengagement	1.39	0.96	1.86	0.98
R^2^	0.13		0.13	
F	4.78, *p* = 0.001		5.01, *p* = 0.001	

Note: PR and G, positive reinterpretation and growth; MD, mental disengagement; RC, religious coping.

## Data Availability

The datasets generated and the final datasets analyzed during the current study are available from the corresponding author on reasonable request.

## Public Involvement Statement

No public involvement in any aspect of this research.

## Guidelines and Standards Statement

This manuscript was drafted against The Strengthening the Reporting of Observational Studies in Epidemiology (STROBE) Statement.

## Appendix A

**Table A1:** STROBE Statement—Checklist of Items that Should be Included in Reports of Observational Studies.

	Item No	Recommendations	Comments
Title and abstract	1	(a) Indicate the study’s design with a commonly used term in the title or the abstract	This is a cross-sectional observational study. Information contained in materials and methods first section.
		(b) Provide in the abstract an informative and balanced summary of what was done and what was found	Information contained in lines 28–46.
Introduction			
Background/rationale	2	Explain the scientific background and rationale for the investigation being reported	Completed. Information is contained on lines 49–108.
Objectives	3	State specific objectives, including any prespecified hypotheses	Information contained in pages 109–112.
Methods			
Study design	4	Present key elements of study design early in the paper	Page 3.
Setting	5	Describe the setting, locations, and relevant dates, including periods of recruitment, exposure, follow-up, and data collection	Pages 3–4.
Participants	6	(a) Cohort study—Give the eligibility criteria, and the sources and methods of selection of participants. Describe methods of follow-up Case–control study—Give the eligibility criteria, and the sources and methods of case ascertainment and control selection. Give the rationale for the choice of cases and controls. Cross-sectional study—Give the eligibility criteria, and the sources and methods of selection of participants	This is a cross-sectional study. People with ID and SMD were identified from case notes in primary care centers. Diagnoses of bipolar disorder, schizophrenia, or severe personality disorder were eligible to participate in the SMD group, and those with moderate or severe ID in the ID group. Carers who were cohabiting with people with ID or SMD were eligible to participate. Information is contained in pages 3, 5.
		(b) Cohort study—For matched studies, give matching criteria and number of exposed and unexposed Case–control study—For matched studies, give matching criteria and the number of controls per case	
Variables	7	Clearly define all outcomes, exposures, predictors, potential confounders, and effect modifiers. Give diagnostic criteria, if applicable	Information contained on page 4.
Data sources/measurement	8 *	For each variable of interest, give sources of data and details of methods of assessment (measurement). Describe comparability of assessment methods if there is more than one group	The data sources described on Setting, and Participants section (pages 3–4).
Bias	9	Describe any efforts to address potential sources of bias	Page 9.
Study size	10	Explain how the study size was arrived at	This was the feasibility study that explored the use of measures and the acceptability of the interviews to the Mongolian caregiving population.
Quantitative variables	11	Explain how quantitative variables were handled in the analyses. If applicable, describe which groupings were chosen and why	Normality test was checked for quantitative variables, and parametric tests were analyzed.
Statistical methods	12	(a) Describe all statistical methods, including those used to control for confounding	Spearman’s correlation test to determine any associations between depression, stress, anxiety, coping styles, and age. We also used independent sample t-tests and chi-square tests to compare the socio-demographic data between the ID and SMD groups, and multivariate analyses of variance (MANOVA) to examine the caregiver’s differences in depression, anxiety, and stress symptoms. Chi-square tests were also used to compare the differences in the use of coping styles by the caregivers and multiple regression analysis to investigate associations between the four coping styles and depression and stress symptoms.
		(b) Describe any methods used to examine subgroups and interactions	
		(c) Explain how missing data were addressed	All missing data were checked and filled out. If unavailable to check, excluded from data.
		(d) Cohort study—If applicable, explain how loss to follow-up was addressed Case–control study—If applicable, explain how matching of cases and controls was addressed. Cross-sectional study—If applicable, describe analytical methods taking account of sampling strategy	
		(e) Describe any sensitivity analyses	
Results			Comments
Participants	13 *	(a) Report numbers of individuals at each stage of study, e.g., numbers potentially eligible, examined for eligibility, confirmed eligible, included in the study, completing follow-up, and analyzed	Information is contained on page 5. Here, 3268 were identified as having SMD or ID in the geographic area and were on the case list of the primary care centers. Of these, 653 had caregivers.
		(b) Give reasons for non-participation at each stage	From the total 160 potential participants who were contacted, 5 were not eligible from the SMD group as we found they were not living with the individual, 25 were unable to complete the questionnaires (10 of which were from the ID group and 15 from the SMD group). Ten were excluded due to missing data. Our 75% response rate is represented in the study flow diagram in Figure 1. This represented 18.7% of the total 653 originally identified.
		(c) Consider use of a flow diagram	Study flow diagram included in Figure 1
Descriptive data	14 *	(a) Give characteristics of study participants (e.g., demographic, clinical, social) and information on exposures and potential confounders	Socio-demographic characteristics of age, gender, and educational achievement between both groups are shown in Table 1. There were statistically significant differences in the age and education levels with the average age of the caregivers in the ID group of 37.66 years (SD = 13.62), compared to 45.23 years (SD = 12.72) in the SMI group. Numbers of men and women were equivocal in groups and between groups (Table 1).
		(b) Indicate number of participants with missing data for each variable of interest	n = 10 were excluded due to missing data.
Outcome data	15 *	Cross-sectional study—Report numbers of outcome events or summary measures	The outcome events included in the Results section.
Main results	16	(a) Give unadjusted estimates and, if applicable, confounder-adjusted estimates and their precision (e.g., 95% confidence interval). Make clear which confounders were adjusted for and why they were included	The results of the association between coping styles and psychological status as depression and anxiety are shown in Table 5. Positive reinterpretation and growth coping style was inversely associated with depression and stress in both groups. Further, religious coping and behavioral coping was positively associated with depression and stress.
		(b) Report category boundaries when continuous variables were categorized	
		(c) If relevant, consider translating estimates of relative risk into absolute risk for a meaningful time period	
Other analyses	17	Report other analyses done, e.g., analyses of subgroups and interactions, and sensitivity analyses	
Discussion			
Key results	18	Summarise key results with reference to study objectives	We found that caregivers of people with ID reported significantly higher levels of depression, anxiety, and stress than those of people with SMD in our samples. The ID carers may therefore be at risk of experiencing psychological distress.
Limitations	19	Discuss limitations of the study, taking into account sources of potential bias or imprecision. Discuss both direction and magnitude of any potential bias	We recognize that the sample size may not have been representative of the overall population of caregivers of people with these diagnoses in Mongolia. Secondly, there was a significant difference in ages between the groups. Age may influence the caregiver’s experience, coping skills, and perceived burden, so a broader study with increased numbers across all age groups would be useful. Lines: 341–369.
Interpretation	20	Give a cautious overall interpretation of results considering objectives, limitations, multiplicity of analyses, results from similar studies, and other relevant evidence	Information contained on pages 8–9.
Generalisability	21	Discuss the generalisability (external validity) of the study results	Information contained on pages 8–9.
Other information			
Funding	22	Give the source of funding and the role of the funders for the present study and, if applicable, for the original study on which the present article is based	This research did not receive any specific grant from funding agencies in the public, commercial, or not-for-profit sectors.

* Give information separately for cases and controls in case-control studies and, if applicable, for exposed and unexposed groups in cohort and cross-sectional studies.
